# Diagnosis and management of people with venous thromboembolism and advanced cancer: how do doctors decide? a qualitative study

**DOI:** 10.1186/1472-6947-12-75

**Published:** 2012-07-20

**Authors:** Miriam J Johnson, Laura Sheard, Anthony Maraveyas, Simon Noble, Hayley Prout, Ian Watt, Dawn Dowding

**Affiliations:** 1 Hull York Medical School, Hertford Building, The University of Hull, Cottingham Road, Hull, HU6 7RX, UK; 2 Department of Health Sciences, The University of York, York, UK; 3 Academic Department of Oncology, Queens Centre for Oncology and Haematology, Castle Hill Hospital, Hull, UK; 4 Department of Primary Care and Public Health, School of Medicine, The University of Cardiff, Cardiff, UK; 5 School of Healthcare, The University of Leeds, Leeds, UK

**Keywords:** Venous thromboembolism, Cancer, Palliative, Clinical decision making

## Abstract

**Background:**

The treatment of cancer associated thrombosis (CAT) is well established, with level 1A evidence to support the recommendation of a low molecular weight heparin (LMWH) by daily injection for 3–6 months. However, registry data suggest compliance to clinical guidelines is poor. Clinicians face particular challenges in treating CAT in advanced cancer patients due to shorter life expectancy, increased bleeding risk and concerns that self injection may be too burdensome. For these reasons decision making around the diagnosis and management of CAT in people with advanced cancer, can be complex, and should focus on its likely net benefit for the patient. We explored factors that influence doctors’ decision making in this situation and sought to gain an understanding of the barriers and facilitators to the application of best practice.

**Methods:**

Think aloud exercises using standardised case scenarios, and individual in depth interviews were conducted. All were transcribed. The think aloud exercises were analysed using Protocol Analysis and the interviews using Framework Analysis.

Participants: 46 participants took part in the think aloud exercises and 45 participants were interviewed in depth. Each group included oncologists, palliative physicians and general practitioners and included both senior doctors and those in training.

Setting: Two Strategic Health Authority regions, one in the north of England and one in Wales.

**Results:**

The following key issues arose from the data synthesis: the importance of patient prognosis; the concept of “appropriateness”; “benefits and burdens” of diagnosis and treatment; LMWH or warfarin for treatment and sources of information which changed practice. Although interlinked, they do describe distinct aspects of the factors that influence doctors in their decisions in this area.

**Conclusions:**

The above factors are issues doctors take into account when deciding whether to send a patient to hospital for investigation or to anticoagulate a patient with confirmed or suspected VTE. Many factors interweave and are themselves influenced by and dependent on each other. It is only after all are taken into account that the doctor arrives at the point of referring the patient for investigation. Some factors including logistic and organisational issues appeared to influence whether a patient would be investigated or treated with LMWH for a confirmed VTE. It is important that services are optimised to ensure that these do not hinder the appropriate investigation and management of individual patients.

## Background

Venous thromboembolism (VTE) such as deep vein thrombosis (DVT) or pulmonary embolism (PE) occurs in 1 in 1000 patients and globally affects 6.5 million people annually [1,2]. It is particularly common in patients with cancer and the incidence appears to increase with disease progression. Approximately one sixth of cancer patients will have symptoms due to VTE [3,4] and one study in advanced cancer patients indicated revealed over half to have evidence of DVT on routine testing [5]. The treatment of VTE usually consists of 3 to 6 months anticoagulation with warfarin [6-12], but this regime is associated with high bleeding complications and recurrent thromboses in cancer patients [13,14]. Three randomised controlled trials have demonstrated low molecular weight heparin (LMWH) to be superior to warfarin in the management of cancer associated thrombosis (CAT) and LMWH is now recommended as the anticoagulant of choice in patients with malignant disease [9,11,12,14-18].

The management of VTE in patients with advanced cancer pose particular challenges for doctors [19,20]. Firstly, the complications of bleeding and recurrent thrombosis are likely to be greater than in the general cancer population. Second the data informing VTE guidelines in cancer patients contained few patients of poor performance status or short prognosis. Finally clinical decision making can be complex in patients for whom the focus of treatment is palliative, but who are not imminently dying [20] since best care will focus on control of symptoms and maintaining quality of life rather than treating a clinical condition at all costs.

Data from a national venous thromboembolism registry (Verityonline) suggest the use of LMWH is not routinely used in cancer patients suggesting a disparity between clinical practice and the evidence [21]

Existing literature has highlighted that knowledge of research evidence is rarely enough by itself to change practice [22,23]. The factors affecting practitioners’ decision making behaviour can vary from individual characteristics (such as lack of knowledge about the evidence), patient factors (complexities associated with advanced disease), through to organisational aspects (such as lack of facilities or equipment). The implementation of evidence based guidelines is often carried out uncritically, with little consideration of the issues which may encourage doctors to change their behaviour, or consideration of implementation strategies that may be the most appropriate for a specific clinical context.

There is currently little evidence examining the factors that influence clinicians’ decision making surrounding the management of patients with advanced cancer and VTE. A vital step towards improving patient care therefore, is to explore how doctors make clinical decisions, and to understand the barriers to and facilitators for the application of best practice guidelines within the context of broader organisational culture and policy initiatives.

In this paper we report the key findings of a study designed to address the following research questions:

1. How do medical practitioners currently make decisions about the treatment of patients with VTE and advanced cancer?

2. What are the barriers and facilitators to change with regard to the implementation of current best practice in patients with advanced cancer and VTE?

3. What strategies would be most effective for implementing best practice evidence in these patients?

## Methods

We addressed the research questions using a combination of think aloud protocols and individual semi-structured interviews. In the first stage we focussed on doctors’ decision-making processes using a think aloud method. In the second stage we investigated the experience of the participants, to explore the barriers and facilitators to good practice in the diagnosis and management of people with advanced cancer and VTE using in-depth individual interviews.

NHS Research Ethics and Research Governance approvals were obtained prior to the commencement of each stage.

### Think aloud study

Think aloud is a method commonly used to explore the cognitive processes used by individuals when making decisions. Study participants were asked to verbalise their thoughts when presented with clinical case scenarios ranging in length and complexity. Think aloud assumes that the subjects’ verbalisations represent the thought processes that they are using whilst making a decision [24]. This approach enabled us to examine the information clinicians used to inform their decisions, together with the nature of decisions taken in relation to the diagnosis and treatment of VTE and cancer.

Six case scenarios were derived for the study by clinicians in the project team (MJ, SN, AM), drawing on their expertise and clinical experience of managing patients with advanced cancer and VTE. Scenarios were presented in the form of a clinical record to represent the information available to the clinician in practice as closely as possible. It is important to make the cases as realistic as possible as clinician performance on a decision task has been found to be ‘task specific’ [24]. The scenarios covered a range of specific decisions and situations in patients with cancer; variation in prognosis, clinical severity of the suspected episode of VTE, age, sex and social circumstance. The scenarios were tested for face and content validity with a panel of clinical experts and modified accordingly, drawn from oncologists, palliative care physicians and general practitioners (n = 4), identified by members of the research team, and their feedback incorporated.

During the think aloud task, participants received a standard introduction to the study by the researchers. This explained the methodology of think aloud, reassured the participants of strict confidentiality and clearly stated that the think aloud exercise was not a test which had ‘right or wrong’ answers. Participants were asked to read the scenarios a few sentences at a time and verbalise their immediate thoughts about the presented case out loud. Additional information about the cases was available from the researcher if requested by the participant. Participants were not asked to define a treatment, as we wished to explore whether treatment for each case scenario would be part of what the participant ‘thought aloud’ using their own decision making process or not. During silences, the researchers prompted participants to keep thinking aloud, but no specific additional direction was given.

### Semi-structured interviews

In-depth interviews were developed from themes arising from the initial analysis of the think aloud data together with a focus on the known barriers and facilitators to practice around VTE and cancer. Questions focussed on barriers to practice for VTE in cancer patients and centred on: anticoagulation; diagnosis & treatment of VTE; logistical, clinical, institutional or attitudinal issues; positive facilitators to their practice. Questioning was adaptive to the responses of the participants.

The topic guide was restructured and amended throughout the fieldwork as new themes emerged.

All participants provided written, informed consent prior to taking part in the study, which included explicit permission for the use of anonymised verbatim quotations in dissemination. Participant anonymity was ensured by the use of study identification number.

#### Sample

Participants for both stages of the study were purposively sampled by specialty (oncology, palliative medicine or GP) and grade, in order to seek diversity of opinion according to experience and focus of practise. These three groups were chosen because all may be involved in the diagnosis and management of patient with advanced cancer and VTE. Although GPs may be less involved with initiating treatment, they will be involved in decisions regarding investigation and continuation of treatment in the community. We recruited participants from two regions of the UK, one in the North of England and the other in Wales. Seventy percent of those taking part in the think aloud study also agreed to an in depth interview; the remainder of the participants who were interviewed were recruited by snowball sampling, to maximize sample variation in the sample. Participants practised across a geographically broad spectrum in both sites, representing both urban and rural areas. Oncologists were recruited from two large teaching hospitals, two oncology hospitals and two district general hospitals. Palliative medicine doctors worked across acute hospitals and a wide range of hospices (some participants had a dual hospital/hospice role).

The sample size in the interview study was determined from previous studies as likely to provide adequate data to gain in-depth insights into clinicians’ decision processes [25].

#### Data collection

Potential participants were identified via registers in the public domain and regional Deaneries. They were sent an invitation letter outlining the purpose of the study and those interested in taking part were contacted for the necessary consent and interview arrangements. Data collection was mostly conducted at the participants’ place of work although a minority of participants were interviewed in their home. Fieldwork was conducted by non-clinical experienced researchers (LS & HP) in order to prevent the “dual role” that can occur when qualitative work is conducted by clinician researchers, and in particular to minimise the risk that participants could perceive the think aloud study as a test of knowledge thus reducing the likelihood that the participants give an unbiased reflection of their actual practice. This was particularly important as three of the research team are considered to be opinion leaders and this may have been known by some respondents. Participants were reassured that all data would be anonymised.

Fieldwork for the think aloud study took place between March and August 2010 and between September 2010 and January 2011 for the interview study. All think aloud exercises and semi-structured interviews were digitally recorded and transcribed for analysis.

#### Data analysis

All data were managed using the software package Atlas-ti.

### Think aloud study

Think aloud transcripts were analysed using protocol analysis to identify the information and specific cognitive processes involved in individual decision making [24,26,27]. Verbalisations were segmented into codes representing elements of identified thought processes; using a coding framework adapted from Twycross & Powls [28] and Lamond et al. [27]. The coding framework was developed by DD, LS and HP and resulted in the following codes: Collect, Interpret, Clinical Evaluation, Social Evaluation, Plan, Reason, Opinion, Diagnose, Goal (Appendix A). Every transcript was coded by LS and then DD and HP each double coded a half of the dataset. All disagreements between first and second coding were marked up and consensus was reached through discussion. Every sentence was coded into one or more of the codes listed above with many sentences having multiple coding attributed to them. The main codes related to reasoning were Plan, Reason & Clinical Evaluate. These codes were closely scrutinised to ascertain doctors decision making processes.

### Interview study

Interview data were analysed using the five stages of Framework Analysis [25] via a process of; familiarisation, identifying a thematic framework, indexing, charting and finally mapping and interpretation. DD and LS independently scrutinised the same six transcripts which were selected for maximum variation. Both individuals devised draft coding frameworks, which were then compared to devise an initial coding framework based on comparable themes. Further iterations of the framework occurred as coding progressed, through discussion between DD and LS and then through the wider forum of the project management group and subsequent revision.

#### Data synthesis

Following initial coding of both elements of the study an initial description of key issues was produced. DD and LS then mapped each of the elements of the individual analyses to identify commonalities and discrepancies in issues and themes arising out the data. A conceptual framework was produced to provide an overview of the data mapping, providing a synthesis of data from both elements of the study.

## Results

Forty six clinicians, aged between 28 – 61 years, (22 women; 24 men) participated in the think aloud study. All three specialties were represented, (15 oncologists; 16 palliative physicians; 15 GPs) and each group had a mixture of senior and junior doctors. Forty five clinicians (26 women, 19 men) were interviewed, ranging in age from 28 to 58 years. Each specialty group had a mixture of junior and senior doctors. More oncologists (n = 20) were interviewed than palliative physicians (n =15) or GPs (n = 10), as data saturation was reached earlier in the latter two groups.

Analysis of the think aloud data identified 11 codes relating to doctors’ engagement in a decision making strategy. The codes reflected different stages of the decision process related to information use, reasoning processes used and the rationale or goal of the decision process (Appendix A). Coding Framework for Think Aloud Interview. Five themes arose out of the analysis of the interview data; 1) logistical & organisational issues (split between investigation & treatment), 2) ethical frameworks & the concept of appropriateness, 3) patient & disease specific factors and 4) knowledge, evidence & experience which doctors used to formulate decisions and 5) VTE risk assessment and prophylaxis.

### Framework overview

Synthesis of the data suggested that there were two distinct decision phases that occur when a clinician is faced with an individual who has cancer and a potential VTE. The first phase involved an assessment of the likelihood of VTE and whether or not to send the patient for a diagnostic test, and if so what test might be appropriate. The second phase, if they decided that the patient did have a VTE, was a consideration whether or not to treat it, and if so, the relative benefits/burdens of LMWH over Warfarin. Factors that influenced the decisions taken included clinicians’ evaluation of the patient’s prognosis (stage of illness), the appropriateness of intervening, logistical and organisational issues associated with intervening and patient’s preferences. The discussion of these factors was multi-dimensional and complex. We used two methods to address the research question, in order to gain insights into the cognitive processes and personal experience of participants. For ease of discussion in this paper we present findings from the think aloud study and the interview study separately discussing each influencing factor in relation to the different decision phases where applicable, before providing a summary of the main issues.

### Think aloud findings

#### Clinical evaluation underpins decisions about investigation and treatment

One of the main cognitive activities engaged in by clinicians was evaluation of the risks from the VTE, possible treatment options of the VTE and the underlying cancer – especially if this was extensive and likely to result in a poor prognosis for the patient. However, opinion was divided about the relative merits of each, and different conclusions regarding management plans were seen. Much of the risk/benefit balance considered was a clinical evaluation, related to the patient’s clinical condition (e.g. likelihood of VTE). However, GPs and palliative physicians were also more likely to take into account the contributory social issues, such as distance to the hospital, and the impact on dependants such as small children; these issues were carefully considered. Summaries of the scenarios referred to in this section can be found in Appendix B. Scenario summaries.

"“So on balance it’s about whether there’s a *risk of bleeding* into her brain tumour I suppose is something to consider but even that *she has a confirmed PE* it will be very difficult not to treat it but it is something that we have to let the patient and the family know that there is a *risk of her bleeding* into the tumour and potentially that will be very detrimental to her life expectancy if she does bleed into the tumour, *her life expectancy is not very good anyway*.” (*Oncology registrar, England, ID17,* scenario 5)"

"“He needs an urgent, urgent scan of his leg, mega urgent and I think *these children are potentially vulnerable* because he could die today. I think this is a mega priority case and basically *he’s got a massive DVT* until proved otherwise and he’s going to need to be anticoagulated and the big problem is the children and he should not be alone either because if he has a massive PE, he’ll die in front of the children when there’s nobody there.” (*Palliative medicine consultant, Wales, ID10,* scenario 2)"

#### Plans for investigation or treatment and their reasons

Interwoven with evaluations of the patient’s condition were plans regarding investigation and treatment and the rationale for that plan (coded as a reason). Although there were similar processes used with regard to weighing up patient risk and benefit, the plans made could vary between doctors. Most variation centred on whether to investigate or not, whether to anticoagulate or not, and if so which anticoagulant to use. The following two doctors from the same specialty, at the same training grade came to opposite conclusions.

"“*There is the risk, somebody with brain mets*, *that if we gave the Clexane there is an increased risk of having a bleed in the brain* but it’s not an absolute contraindication because there is nothing to say that on the scan there were signs of bleeding, so I think given that she’s now acutely unwell with confirmed bilateral pulmonary emboli, *I would, from a best interest point of view, see treating those as a priority over the risk of the brain mets bleeding really.*” (*Palliative medicine registrar, Wales, ID8,* scenario 5)"

"“*I don’t think I would recommend anticoagulation really.* I don’t think it’s likely to help her. I don’t think it will prolong her life probably. I don’t think it will particularly help her symptoms either… possibly make her bleed into her brain.” (*Palliative medicine registrar, England, ID5,* scenario 5)"

The scenarios generated differing opinions as to which anticoagulant to use. In all scenarios, if anticoagulation was planned, warfarin was more likely to be chosen by GPs than by oncologists or palliative physicians. All the palliative physicians and most of the oncologists planning to anticoagulate planned to use LMWH alone. A minority of oncologists planned to use initial LMWH followed by warfarin. Reasons for choosing LMWH included the ease of control of anticoagulation and knowledge that it is more effective in cancer.

"*“*In terms of how I would anticoagulate her, I would be inclined with this sort of patient to probably go with low molecular weight heparin rather than Warfarin, it’s just more controllable…” (*Oncology consultant, England, ID14*, scenario 1)"

"“and she’s got cancer and it’s been shown in multiple studies that patients with cancer undergoing treatment either do better or it’s safe to give them low molecular weight heparin” (*Oncology consultant, England. ID 33*, scenario 1)"

Again in scenario 2, GPs were more likely to think of using warfarin than the other doctors.

"“But if he refuses to go for investigation for his own self and he’s able to weigh up the information and come to an opinion and he has got a capacity to make that decision, from his point of view, I’ve got no choice but to start him on Warfarin” *(Senior GP, England, ID28,* scenario 2)"

In addition, some oncologists and palliative physicians specifically rejected warfarin as a choice because of the presence of liver metastases. No GP rejected warfarin in scenario 2.

"“He needs to be started on a low molecular weight heparin today and with his liver metastases I would thinking, forget warfarin and just keep him on a low molecular weight heparin indefinitely” (*Palliative medicine consultant, England, ID4*, scenario 2)"

However, in scenario 6, about a patient in the last few days of life, no doctor recommended warfarin, and the decision-making then centred on the potential benefits, burdens and practicalities of LMWH in this situation.

"“…avoid injections of Clexane which are in themselves unpleasant because they have to be subcutaneous, they can hurt, they can cause bruising, discomfort in the abdomen”. (*Oncology registrar, England, ID19*, scenario 6)"

"“The fact that his oral intake is variable and he’s having difficulty swallowing solids also complicates your choice of anticoagulation, he may not be able to swallow Warfarin” (*Senior GP, England, ID 24*, scenario 6)"

Throughout most scenarios, there was a strong emphasis on the importance of discussing patient wishes (nearly three quarters of respondents) as well as a willingness to take advice from other colleagues.

"“I mean I think that the patient’s first statement is, you know, “do whatever” but I actually think that it is really important that he is involved in the decisions here because he’s very much sort of towards end of life now and I wouldn’t recommend for readmission to hospital and acute treatment of the DVT” *(Senior GP, Wales, ID1,* scenario 6)"

"“So your first question to this patient is, how much do you want me to do? He’s not refusing all medical treatment because he’s having prescribed medicines, so it may well be that he will consider anticoagulation if a DVT was confirmed, so I would have that first discussion with the patient about whether he would want me to investigate or not because if he wasn’t going to accept any treatment, then clearly there would be no point in proceeding.” (*Oncology consultant, Wales, ID12,* scenario 6)"

"“I’m thinking this is really difficult and there is the children involved as well so that does make a difference about whether you would want the social work team to be involved and I’d probably speak to my consultant and maybe a surgeon. Just to find out how risky it is to put someone on a treatment dose of tinzaparine when they’ve had hepatic surgery two weeks ago” *(Palliative registrar, England, ID3,* scenario 2*).*"

The latter quote also demonstrates how the patient’s circumstances and the impact of management options may affect their dependants, are taken into account.

### Interview findings

Five themes were derived from the interview data; 1) logistical & organisational issues (split between investigation & treatment), 2) ethical frameworks & the concept of appropriateness, 3) patient & disease specific factors 4) knowledge, evidence & experience which doctors used to formulate decisions and 5) VTE risk assessment and prophylaxis. As the research questions related to the *diagnosis and management of established VTE*, the findings relating to risk assessment prophylaxis will be reported elsewhere. The first two themes were key factors influencing decisions and will be discussed under main headings. The impact of themes (3) and (4) -patient &disease factors, and the evidence &experience of doctors, is mediated through both of the first two themes. In view of this, aspects of themes three and four will be discussed under the two main headings – logistical and organisational issues and the concept of appropriateness

### Theme 1: Logistical & organisational issues

#### Investigation

The burdens of investigation resulting from logistical and organisational factors, seemed to be a particular problem for patients with advanced disease, e.g. although long waits in radiology departments, clinics or emergency rooms or unnecessary days in hospital are frustrating for people with better performance status, they may be better able to cope with the accompanying physical demands than those with poorer performance status. There were particular issues related to how easily accessible the services were for diagnostic tests in particular. This included how far the patient had to travel, availability of transport and the availability of ‘same day’ diagnostic test facilities. If the patient was likely to have to stay overnight in a hospital just to get a particular test completed for instance, the doctor may not feel that it was an appropriate choice for them, taking into account other factors (discussed in further sections).

"“… in a hospice that’s 3 and a bit miles away from the local hospital is that if I have somebody here who might have a DVT or a PE and they’re fit enough to be investigated and treated, I can’t always access a service which will diagnose VTE the same day. If I send the patient up to the hospital accepting that the patient may stay overnight, there is an issue that perhaps it won’t be see as urgent because they’ve arrived and they go in the queue like everybody else and they might end up staying there 3 or 4 days when their life expectancy is quite short and I don’t want them to be spending and they particularly don’t want to spend time in hospital.” *(Palliative medicine consultant, England, ID18*)"

"“The main difficulty with ambulance transport is the time that it sometimes takes… and quite often things get escalated to a 999 ambulance because there aren’t any acute ambulances. So if people haven’t got their own transport and nobody to take them or you feel they’re too unstable, that’s rare because we’re a mile away from the hospital, usually it’s quicker for people and safer for people to go straight there in private transport than by ambulance because of the difficult patient transport because of the time it takes.” *(Senior GP, Wales, ID 31)*"

In some areas there were examples of streamlined practice which minimised disruption to patients. These included specialised services for testing for VTE, and the employment of specialist nurses who co-ordinated the VTE and thromboembolism services across NHS organisations.

"“What we’ve done is reconfigured the in-patient base … so I could have said, “well go to [town] Casualty” and I know that if I send that person to [town] Casualty anything could happen because you get a junior doctor not knowing they’ve got cancer or whatever and probably admitted to a geriatric ward where 3 days later they do a Doppler ultrasound and get confused and muddled by all manner of the rest of her case versus “oh just tell her to come to [assessment unit].” I’ve rung [assessment unit] and when she gets to [assessment unit], I said “send her straight down for a Doppler ultrasound and if it’s negative send her home and if it’s positive put her on tinzaparin and send her home.” *(Oncology director, England, ID5)*"

"“I do a clinic on a Thursday over in the main department at [hospital] and there is a little laminated sheet stuck on the x-ray box and it says, *Suspect a DVT? Call this number* and they do one straight away. Basically if somebody comes in to your clinic with a swollen painful leg, you ring that number, you whizz them round to x-ray and you get a Doppler there and then, I mean it’s a 9 to 5 service it’s not out of hours, but yeah it’s easy …. “*(Oncology consultant, England, ID24)*"

"“I know some places will have a specialist nurse who deals with these kind of calls and then organises and liaises with the radiologist services directly and they have access to that but they aren’t coming through the registrar on call handling x number of other calls, so that takes a bit of a burden off us.” *(Oncology registrar, England, ID10)*"

The complex balance of the risk of bleeding against the risk of further VTE (if that is the correct diagnosis) leads to an urgency with regards to arranging an investigation for some patients. Not all doctors had access to streamlined investigations and although urgent scans could be arranged, it took considerable time, often from the consultant, because this had to be arranged personally.

"“… there was a high risk of him having a PE but then also he has a bleeding in his duodenum so I was between a rock and a hard place, if I now anticoagulate him then I might set off a significant gastrointestinal bleed and if I don’t anticoagulate him and he really is positive for a PE and he might die from this PE so you are between a rock and a hard place. But then *you overcome that by walking down to the CT department* and telling them the story and then they put him on the list as soon as ever and you make a clinical decision… *and sometimes in terms of time management that is a little bit difficult*.” (*Oncology consultant, England, ID28*)"

### Treatment

Organisational difficulties were also a concern when optimising treatment, for example, a hospice physician or oncologist may initiate LMWH, but problems arise subsequently with ongoing prescription and monitoring by the GP.

"“But I think it’s important that there is good coordination around that because normally what will happen is the GP will suddenly find out, oh the patient is on anticoagulation. But it depends who they’ve seen in the hospital, it also may not be as clear as to why they were started on it, how long they need to be on it, what dose they were started on.” *(Oncology consultant, England, ID12)*"

Clear tension between the two settings was evident in some areas. Interview data from oncologists demonstrated a perception that this may be partly cost-driven (warfarin is cheaper than LMWH) and was a variable problem depending on whether the prescribing budget responsible for LMWH given to patients in the community was hospital or community. Difficulties with community administered LMWH were also discussed by respondents.

"“They [GPs] don’t know how to handle VTE in cancer patients as well as I do… all I will be able to do at that point, is make it absolutely clear what the specialist advice is and respect the fact that if something goes wrong, I will put my hands up and say, well I made it quite clear that I didn’t want this patient to be on warfarin and the GP will then have to defend their decision. It will be a very sad when they stand up and say, “well I did this because it was cheaper”.” *(Oncology director, England, ID5)*"

"“I’ve had one patient who nearly didn’t get treatment over a bank holiday weekend because the GP was refusing to prescribe it … we finally managed to get them a few days’ supply from the GPs. So they almost didn’t get treated over that weekend. But apart from that normally it’s just inconvenience rather than another dangerous thing like that…It’s happened 2 or 3 times though in the last year and that’s just me. I think other consultants have had problems.” *(Oncology consultant, Wales, ID22)*"

"“They can rush around all day long district nurses doing tasks, taking bloods, giving [LMWH] injections. They can’t be doing everything else…all the other things that district nurses can’t do if they spend three quarters of an hour driving to a patient giving an injection, driving back, everyday. That’s actually a massive cost, not financial cost. Cost in terms of real limited number of nurses. If relatives and patients can be taught to give injections themselves, I would favour that because I think it’s massive, massive if you really live somewhere rural.” *(Palliative medicine doctor, England, ID6)*"

### Theme 2: The concept of appropriateness

The concept of appropriateness; whether doctors considered it ethically appropriate to investigate or treat the patient for VTE, was a major theme arising from the interview data. In this paper we provide an overview of the key issues arising from this theme. As it was an important and complex area of debate we will be reporting the results of this theme in detail subsequently.

The concept of appropriateness was inextricably linked with expected patient prognosis and availability of appropriate services; doctors were more willing to investigate and treat those with a good prognosis or where services were easily accessible. There was debate about how appropriate it was for LMWH to be administered to a patient thought to be in the last few days/weeks of life and if these patients should be moved for investigation.

"“I guess the other issue, which I don’t know if it’s a barrier particularly, but it’s certainly a poorer prognosis end of things, how appropriate it is to treat problems, particularly in somebody who is approaching end of life care and certainly in the last few days of life.” *(Oncology consultant, England, ID7)*"

"“So it’s not that they’re nearing the end of life because of a clot, they’re nearing the end of life because of their disease. If that’s happening anyway you have to think about the burden of the investigation and the treatment*” (Palliative medicine consultant, Wales, ID 25)*"

The importance of assessing each patient’s situation individually with regard to likely benefit within the expected prognostic timeframe, balancing against unnecessary interventions in the last few hours/days of life was highlighted. For example, if a patient has had benefit from anticoagulation, or has had recurrent problems with VTE, then the threshold for treating would be lower for longer.

"“But I think it is again very hard to sort of say, to come up with a general rule. …, if they seem to be getting recurrent PEs or they’ve had very difficult to manage sort of swollen limbs as a result of thromboembolic problems etc., that has in the past appeared to be managed better by actively managing it, then that would influence you to give it a go until such time that it became apparent that it was no longer appropriate if they were in the sort of unconscious phase, you know, dying within hours to short days*.” (Oncology consultant, England, ID 18)*"

### Summary of main issues

We set out to explore the influences affecting the way doctors made clinical decisions in this complex clinical situation, and to discover the barriers and facilitators to best practice, in particular, with regard to the prescription of LMWH rather than warfarin.

We identified two main areas of decision making in our data which related to the investigation and management of VTE. Within these decisions, the impact of organisational factors, the considered appropriateness of an intervention (investigation or treatment), estimated patient prognosis and the perceived benefits/burdens of LMWH compared with warfarin influenced doctors’ decisions. Barriers and facilitators to implementation of treatment guidelines were demonstrated within all these influences.

## Discussion

A range of issues were reported by participants to influence their diagnosis and management of VTE in people with advanced, unless the patient was considered to be imminently dying, in which the decision making appeared to be more straightforward.

Many of these factors interweave and are themselves influenced by and dependent on each other. It is only after all are taken into account, resulting in an assessment of whether any further action is *appropriate* for that individual patient, that the doctor arrives at the point of referring the patient for investigation or starting empirical treatment with LMWH.

### Barriers and facilitators to evidence based practice

#### Logistical and organisational

There were a number of logistical and organisational factors that influenced whether or not patients with cancer and suspected VTE were investigated, as well as whether or not they subsequently received treatment.

#### Investigation and diagnosis

Issues relating to the practicalities of investigation and treatment were considered a problem, this may result in the patient not being investigated at all, or being given empirical LMWH, thus exposing the patient to the risk of bleeding for a condition they might not have. At present, smooth streamlined services do not appear to be consistently provided across the areas explored, and the brunt of the adverse effects are likely to be borne by these vulnerable patients with advanced disease who already have a multitude of problems. The decision to investigate and treat patients who would benefit should be unaffected by inadequate organisation of the health care system.

Examples of good practice, where oncology centres had the process of organising investigations, results and treatment showed how the impact of these aspects could be minimised. However, these service improvements are not consistent and there is little in the published literature regarding successful service models in this field. In addition, there was no example where the problems arising from ambulance transport inflexibility had been overcome.

A variety of factors may act as barriers or facilitators to the implementation of service improvements; financial incentives or penalties, media interest, competing priorities and the presence of local champions.

#### LMWH prescription and monitoring

Participants in our study referred to LMWH prescription more often than we had anticipated from national figures of long term LWMH, however, this may be due to the regional influence of SN, MJ and AM who were mentioned in some interviews as opinion leaders, and this may not be reflected across the UK.

Good systems between primary and secondary care are important. It was noted that warfarin was sometimes prescribed in the community. This use of a treatment largely superseded by LWMH may be because GPs had less experience of cancer-related VTE, seeing far fewer patients per year compared with palliative physicians and oncologists. This problem is likely to be more than merely a lack of knowledge, and nationally, the low level of LMWH prescribing is unlikely to be solely explained by GP prescription of warfarin. Evidence based protocols for the funding and shared clinical responsibility between secondary and primary care for therapeutic interventions which may not be initiated in primary care, but for patients whose care is largely in the community may be helpful in resolving the tension. Given that these patients may also be seen by hospice services, it is imperative that clear boundaries of prescribing and monitoring responsibility are set. Examples of shared care protocols regarding LMWH do exist [28,29] but again, this is not consistent.

### Implications for practice and service delivery

Our study findings suggest a number of service developments that might improve adherence to current guidelines and improve quality of care. Patients with advanced disease need to be able to access appropriate clinical tests in a timely manner. Examples of innovative diagnostic and management services should be widely shared, especially same-day diagnostic services. Ambulance services could be given feedback about the need for flexibility, and swift, timely transportation and the availability and funding of DVT nurse specialists to streamline diagnostic and treatment processes encouraged.

Good practice with regard to shared care prescribing and monitoring protocols for LMWH treatment across primary and secondary care settings should be supported and disseminated so that patients will not be prescribed warfarin inappropriately on the grounds of cost or inexperience and lack of knowledge. Change in practice is a complex issue and it is perhaps not surprising therefore, that simply issuing guidelines has not been successful in achieving high rates of prescribing of LMWH. For the most part, we have not found major deficits in knowledge – which guidelines might help. Rather, we have found a complex interplay of structural, process and patient/physician factors which may also need to be considered if appropriate management of VTE is to be achieved.

#### Future research

This study did not directly seek the opinion of patients themselves. It is therefore vital that this is explored to find out what patients consider to be important with regard to successful management of VTE. The development of patient relevant outcomes is key to any further research in this area.

It is clear from our data, that many of the difficulties in decision making come from the fact that the evidence base underpinning the current management guidelines was developed in patients with a much greater performance status. Thus it can be difficult to extrapolate that information to inform the management of the person attending in clinic. Hence it is important to address the evidence gaps in this area of VTE research in order to provide more specific guidance for clinicians managing palliative care patients, including gaining an understanding of the natural history of VTE in people with advanced disease so that the risk-benefit balance of LMWH anticoagulation may be better assessed. Further research into whether, and how, patients are involved in decision making about the diagnosis and management of VTE would also be helpful.

### Limitations

The purpose of the think aloud is to reveal cognitive activities in handling the individual case scenarios and thus we were able to look only at one set of decisions at a point in time. An exploration of how decisions evolve over time would be interesting, but need different types of case scenarios.

Doctors volunteering to participate in this study may be those with more interest in this topic for several reasons: previous experience of difficulties, a raised awareness of the issue as a result of working in the same geographical area as a co-author (SN in Wales, MJ and AM in the North of England), an approach to clinical practice that is committed to contributing to the evidence base, and therefore may not be representative. As the pool of potential oncologists and palliative physicians was far fewer than the GPs this is likely to be a particular issue for the GP group, and may be one reason why some of the accounts from oncologists regarding conflict with GPs over management with long-term LMWH are not mirrored in the GP interviews. Although GPs were the professional group more likely to use warfarin rather than LMWH, the GP participants in the study often referred to taking the advice of oncologists over the patient’s management. It is possible that even if the participant accepted that the think aloud was not a test, they may have revised the subject of venous thromboembolism and cancer prior to the interview.

If one of the co-authors was known to a participant, it is possible that they may have responded with what they thought was the “correct” answer if this differed to their usual practice. The use of a non-clinical researcher and anonymity were used to reduce this risk. Furthermore, to ensure our promise of strict confidence, co-authors did not have access to complete transcripts of interviews conducted with their colleagues. This was to ensure that colleagues could not be identified by personal opinions or clinical examples given which may allow possible identification of participants by co-authors to occur.”

However, none of these issues affect the importance of the experience of the doctors interviewed, and as many of the same issues were raised in both of the geographical areas sampled in this study it is likely that they mirror problems encountered nationally.

## Conclusions

Doctors find making decisions about the diagnosis and treatment of people with VTE and advanced cancer complex, with little published data based on these patients to guide practice. Complexities arise because of the risk-benefit balance of anticoagulation in patients who are at high risk of recurrent VTE and bleeding. This requires a judgement of appropriateness in the context of benefits and burdens within the timeframe of estimated prognosis. Influences on such judgements are inherently contextual and individual both to the patient and doctor.

Logistical issues regarding diagnosis (arranging investigation, transport, avoidance of inappropriate time in hospital) and management (prescribing, monitoring and budgetary responsibility for LMWH, cross-sector team working) add to the complexity. Given the barriers to and the complexity of decision-making that we have found in this area, simple rejoinders to follow evidence are unlikely to succeed in isolation. If we want to improve practice we need to develop carefully considered strategies that address the barriers which result, not only from lack of knowledge, but also from poorly organised services and inadequate understanding of patient priorities for the management of VTE in advanced cancer.

## Appendix A. CODING FRAMEWORK FOR THINK ALOUD INTERVIEWS

### Principles

1. Coding generally to take place at the level of the sentence.“you need to check what her full blood count is to see if she is anaemic and you maybe able to improve her breathlessness by treating that” would be coded as both plan and reason but coded separately this would be:“you need to check what her full blood count is to see if she is anaemic”(Plan)“and you maybe able to improve her breathlessness by treating that” (reason)

2. Double or triple code rather than split up sentences to keep context intact. Examples:

3. When obtaining the retrieved segments at the end of analysis, the ‘reason’ example given above will be meaningless without the plan with which it relates to

4. Two or more of the same operators in the same sentence are to be coded differently if it’s an unrelated topic.

5. A significantly longer sentence than usual can be coded as parts of the sentence

## Codes

1. COLLECT

Questions which the participant asks to inform their thinking:Statements where the participants requests more information:

2. INTERPRET

Stating what the information infers / means, clinicallyRephrasing of the data

3. EVALUATE (Clinical or social)

Making clinical judgements based on the data that is not a diagnosis or goalMaking social judgements based on the data that is not a diagnosis or goalThe difference between Interpret and Evaluate – when participants are interpreting, they are usually doing something basic and simple with the data presented to them but Evaluation is one step further on in that they are saying something about the data which is a judgement. Here is a good example:The first half of this sentence is Interpretation. The participant has taken from the scenario that the patient is 2 weeks after a major operation. The second half is Evaluation as “so he actually shouldn’t be driving himself anywhere” is a social judgment about the patient’s right to drive so soon after having a major operation.There are two distinct processes in reasoning:Interpreting – where the respondent is looking at the information and interpreting it to make sense of it.Evaluate – where the respondent is taking the information and making some form of judgement/diagnosis on the basis of it

4. DIAGNOSE

A firm statement which pinpoints a specific diagnosis.Include any medical diagnosis, does not specifically have to be VTE or cancer related

5. OPINION

Participant offers their own personal opinion on scenarioOr how they would act if in patients position

6. PERSONAL KNOWLEDGE

Procedures / tests / symptoms etc. which participants’ state they know aboutTheir previous experience with other patients:Expression of academic / clinical knowledge which is not interpretation or evaluation. Quotation of study results or statistics:

7. PLAN

Any future plan related to management or treatment.Include discussing future treatment with the patientInclude speculative plans

8. REASON

Any verbalisation which relates to why a participant has chosen a course of actionNOT related to the reason why they have stated a specific diagnosis.NOT related to the reason why they think a patient may have chosen a course of action.

9. PREDICT

What may happen if the participant chooses a course of action or what may happen if they do nothing

10. GOAL

The final aim of all the participant’s planning. Would usually be related to anticoagulation but could be to send patient for a scan/ test or to refer to another clinician e.g. consultant for trainees or oncologist for GPs etc.

11. BARRIERS re LMWH.

As this is the central focus of the study, these need to be noted. These barriers need to be flagged up to inform the topic guide for part 2 so it’s essential we get to them straight away.Include barriers to generic “anticoagulation”Include comments re warfarin if relevant as will help with topic guide

## Appendix B. Scenario summaries

### Scenario 1

A 68 year old single lady has endometrial carcinoma. She has mild but continuous vaginal bleeding. She feels she would not be able to cope if the bleeding became any worse. She presents to the clinic with a 3 month history of progressive breathlessness such that she now cannot walk across her small lounge to the bathroom without severe breathlessness. A ventilation-perfusion scan shows multiple pulmonary emboli.

### Scenario 2

A 38 year self employed man has colorectal cancer with recently resected hepatic metastases. His wife is away at the moment and he is at home with a 3 year old and a 5 year old. His sister has agreed to come up and help on Friday (it is now Monday). He lives in a small village “off the beaten track”, has not been there very long and doesn’t know the neighbours well.This morning he woke to find his left leg swollen and painful, making it quite difficult to walk. He took a taxi to the surgery as his leg was too painful to let him drive, bringing the children with him. He will not leave the children with anyone else (even if he could find anyone else) so is refusing to go for investigation or admission. He will only leave the children once his sister comes on Friday.

### Scenario 5

A 69 year old lady has an extensive small cell lung cancer with newly diagnosed brain metastases. She is due to receive palliative whole brain radiotherapy the following week but now presents with sudden onset breathlessness and pleuritic right side chest pain.On examination she is short of breath at rest with a tachycardia of 100, BP 100/76, her JVP is raised and she is hypoxic on air. A CTPA shows extensive emboli in left and right pulmonary arteries.

### Scenario 6

A 52 year old man has extensive unresectable oesophageal cancer with hepatic and bone metastases. He now spends all day in bed. During a recent hospice admission he was found to have a corrected calcium of 3.4 mmol/l and was successfully treated with intravenous fluids and bisphosphonate. A syringe driver was commenced at the hospice and continues since oral intake is variable. He has developed a painful swelling in his left leg.He is cachectic with sacral oedema. Cardiovascular and respiratory examinations unremarkable but abdominal examination reveals tender hepatomegaly with a small amount of ascites. His left leg is grossly swollen, tender and erythematous to the thigh.He is lucid but weak, saying, “Do whatever you think is best doctor”. Has previously stated he would like to die at home.

## Competing interests

SN, AM and MJ are co-directors of the TRAD Alliance. The TRAD Alliance is supported by an unrestricted educational grant from Pfizer. SN has given lectures on behalf of Pfizer, Sanofi Aventis, Leo Pharma and Boeringer Ingelheim; all fees are donated directly to charity. SN has also received research grant funding from Pfizer. AM is an advisory board member for Leo and for Pfizer and has received research grant funding from Pharmacia/Pfizer. There are no conflicts of interests declared from DD, LS, HP, and IW.

## Authors’ contributions

MJ conceived the idea for the study, participated in the study design, co-ordination, advised on data analysis and drafted the manuscript. DD conceived the idea for the study, participated in study design, co-ordination, and drafted the manuscript. LS & HP collected all the data. LS, HP & DD analysed data. LS co-ordinated the study and interpreted all data. SN, IW and AM participated in the study design, trial coordination and commented on manuscript drafts. MJ, DD, SN, AM and IW were applicants on the original grant. All authors were members of the project management group and approved the final manuscript.

## Pre-publication history

The pre-publication history for this paper can be accessed here:

http://www.biomedcentral.com/1472-6947/12/75/prepub
